# Revisiting Suppression of Interspecies Hybrid Male Lethality in *Caenorhabditis* Nematodes

**DOI:** 10.1534/g3.117.039479

**Published:** 2017-02-16

**Authors:** Lauren E. Ryan, Eric S. Haag

**Affiliations:** Department of Biology, University of Maryland, College Park, Maryland 20740; Biological Sciences Program, University of Maryland, College Park, Maryland 20742

**Keywords:** Haldane’s rule, X chromosome, hybrids, Genetics of Sex

## Abstract

Within the nematode genus *Caenorhabditis*, *Caenorhabditis briggsae* and *C. nigoni* are among the most closely related species known. They differ in sexual mode, with *C. nigoni* retaining the ancestral XO male–XX female outcrossing system, while *C. briggsae* recently evolved self-fertility and an XX-biased sex ratio. Wild-type *C. briggsae* and *C. nigoni* can produce fertile hybrid XX female progeny, but XO progeny are either 100% inviable (when *C. briggsae* is the mother) or viable but sterile (when *C. nigoni* is the mother). A recent study provided evidence suggesting that loss of the *Cbr-him-8* meiotic regulator in *C. briggsae* hermaphrodites allowed them to produce viable and fertile hybrid XO male progeny when mated to *C. nigoni*. Because such males would be useful for a variety of genetic experiments, we sought to verify this result. Preliminary crosses with wild-type *C. briggsae* hermaphrodites occasionally produced fertile males, but they could not be confirmed to be interspecies hybrids. Using an RNA interference (RNAi) protocol that eliminates any possibility of self-progeny in *Cbr-him-8* hermaphrodites, we found sterile males bearing the *C. nigoni* X chromosome, but no fertile males bearing the *C. briggsae* X, as in wild-type crosses. Our results suggest that the apparent rescue of XO hybrid viability and fertility is due to incomplete purging of self-sperm prior to mating.

Interspecies hybrids can provide insight into the genetic mechanisms behind the diversity of organisms, speciation, and the arising of novel traits. Reproductive barriers limit gene flow between species and can be pre- or postzygotic. Prezygotic barriers include behavioral isolation or gametic incompatibility. Postzygotic reproductive barriers include hybrid lethality (at any prereproductive developmental stage) or sterility. It is this latter barrier that is the focus here. The Bateson–Dobzhansky–Muller model proposes a genetic basis for hybrid incompatibility, whereby incompatibilities between heterospecific loci result in impaired function or nonfunction (Dobzhansky 1936). In addition, interspecies hybrids often manifest Haldane’s rule, where the heterogametic sex is more severely impacted, presumably because of sex chromosome hemizygosity (Orr and Turelli 2001). Darwin’s corollary to Haldane’s rule is also observed in many cases, where there is asymmetric male viability in reciprocal crosses (Turelli and Moyle 2007).

*Caenorhabditis* nematodes are an excellent system to study the genetic basis of reproductive diversity. The genus contains both gonochoristic (male/female) and androdioecious (male/hermaphrodite) species, making it possible to study the variation of reproductive mode. The essence of hermaphroditism is limited spermatogenesis in the context of the XX female ovary. How development of the bisexual germline is regulated has been studied heavily in *Caenorhabditis elegans* (*e.g.*, Doniach 1986; Goodwin *et al.* 1993; Ellis and Kimble 1995; Francis *et al.* 1995; Zhang *et al.* 1997; Chen *et al.* 2000; Clifford *et al.* 2000; Luitjens *et al.* 2000) and, more recently, in *C. briggsae* (Chen *et al.* 2001, 2014; Hill *et al.* 2006; Guo *et al.* 2009; Hill and Haag 2009; Beadell *et al.* 2011; Liu *et al.* 2012). These two selfing species, while superficially similar, evolved self-fertility independently (Kiontke *et al.* 2011) and it was achieved via distinct modifications of the global sex determination pathway (Hill *et al.* 2006; Guo *et al.* 2009; Hill and Haag 2009; Chen *et al.* 2014).

The first studies comparing sex determination in hermaphrodite and female relatives of *C. elegans* used candidate gene approaches (de Bono and Hodgkin 1996; Kuwabara 1996; Streit *et al.* 1999; Haag and Kimble 2000; Haag *et al.* 2002; Hill *et al.* 2006; Hill and Haag 2009; Beadell *et al.* 2011; Liu *et al.* 2012) and forward genetic screens (Hill *et al.* 2006; Kelleher *et al.* 2008; Guo *et al.* 2009, 2013). The *C. briggsae* and *C. nigoni* system has opened the tantalizing possibility of using hybrids between them to identify factors distinguishing hermaphrodite and female germline sex determination (Woodruff *et al.* 2010). However, these efforts have been thwarted by extensive genetic incompatibilities. *C. briggsae* × *C. nigoni* hybrids are subject to both Haldane’s rule and Darwin’s corollary to Haldane’s rule (Woodruff *et al.* 2010; Kozlowska *et al.* 2012). Specifically, no viable male F1 are found when wild-type *C. briggsae* hermaphrodites are mated with *C. nigoni* males, but viable yet sterile males are produced when *C. nigoni* females are crossed with *C. briggsae* males. Surprisingly, after laying a few hybrid progeny, *C. briggsae* hermaphrodites mated with *C. nigoni* males are sterilized by the aggressive *C. nigoni* sperm (Ting *et al.* 2014).

F1 females from both possible *C. briggsae* × *C. nigoni* crosses produce viable progeny only when backcrossed to *C. nigoni* (Woodruff *et al.* 2010). This has allowed introgression of marked *C. briggsae* chromosomal segments into *C. nigoni* (Yan *et al.* 2012; Bi *et al.* 2015). These segments remain large in spite of multiple backcrosses, and harbor a number of inviability and sterility loci, some of which impact germline small RNA pathways (Li *et al.* 2016). In addition, polymorphisms within *C. briggsae* and *C. nigoni* can impact the severity of F1 hybrid phenotypes (Kozlowska *et al.* 2012).

As for its ortholog in *C. elegans* (Phillips *et al.* 2005), the *C. briggsae high incidence of males 8 (him-8)* gene is required for faithful segregation of the X chromosome during meiosis (Wei *et al.* 2014). This, in turn, greatly elevates the spontaneous production of XO self-progeny, which are male. Thus, while an unmated *C. briggsae* hermaphrodite will produce < 1% males naturally, *Cbr-him-8* mutants are Him, producing ∼15% males (Wei *et al.* 2014). Recently, Ragavapuram *et al.* (2016) reported that loss of *Cbr-him-8* function can rescue both lethality and sterility in male hybrids bearing the *C. briggsae* X chromosome. As such males might allow F1 intercrosses and new backcross types, we sought to verify and extend these results. Surprisingly, using the same strains as Ragavapuram *et al.* (2016) but with methods that eliminate the possibility of males arising from selfing, we find no evidence of hybrid male rescue.

## Materials and Methods

### Strains

*C. briggsae* PB192 (*Cbr-him-8*(*vI88*) *I*; *stls20120*[*Cbr-myo-2p*::*GFP* + *Cbr-unc-119(+*)] *X*) was provided by Scott Baird (Wright State University OH). *C. nigoni* JU1422 (inbred derivative of wild isolate JU1375) was provided by Marie-Anne Félix (Ecole Normale Supérieure, France). *C. nigoni* wild isolate EG5268 was the gift of Michael Ailion (University of Utah, UT; currently at University of Wisconsin, Madison, WI). All strains were maintained on 2.2% NGM agar (Wood 1988) with OP50 *Escherichia coli* bacteria as food source.

### Cbr-fog-3 RNAi

A *Cbr-fog-3* template for *in vitro* transcription was made with the polymerase chain reaction (PCR) using Taq DNA polymerase (New England Biolabs) with recommended concentrations of dNTPs and primers. Primer sequences (including the underlined T7 promoter required for subsequent *in vitro* transcription) are: forward: 5′-TAATACGACTCACTATAGGGAGCCGACGAAGTTCTTGAAA-3′; reverse: 5′-TAATACGACTCACTATAGGGCCCACCATGGTCTGCAGATC-3′. The PCR product was purified using a QIAquick PCR purification kit (QIAGEN). Next, 780 ng of *Cbr-fog-3* PCR product was used as template in a Megascript T7 *in vitro* transcription reaction (Thermo Fisher Scientific) following the provided protocol. The resulting dsRNA was purified by ammonium acetate and ethanol precipitation. PB192 hermaphrodites were picked onto a separate plate at the L4 stage 12 hr prior to injection. Forty adult worms were mounted on agar pads and injected with *Cbr-fog-3* dsRNA at a concentration of 3000 ng/µl. Injected animals were moved to individual NGM plates seeded with OP50 *E. coli* plates after 8 hr of recovery time.

### Crosses

For interspecies crosses not employing *Cbr-fog-3*(*RNAi*), PB192 hermaphrodites were purged by several days of serial transfer to NGM plates, similar to the method of Ragavapuram *et al.* (2016).

*C. briggsae* mothers who could not self-fertilize were produced through RNAi targeting of *Cbr-fog-3* (Chen *et al.* 2001). Progeny of injected PB192 mothers that appeared to have the feminization of germline (Fog) oocyte stacking phenotype after reaching adulthood were moved in small groups to new agar plates seeded with OP50 *E. coli*, and allowed to sit for 6 hr to ensure that no self-progeny were produced. *C. nigoni* EG5268 males were added at a 2:1 ratio of males/hermaphrodites and allowed to mate for 4 hr. All plugged animals were moved to individual plates. *Cbr-fog-3*(*RNAi*) PB192 pseudofemales were also crossed to *C. briggsae* AF16 males to verify that they had normal fecundity.

### Microscopy

Routine maintenance and crosses were performed using a Leica MZ125 stereoscope. Analysis of male germline morphology used differential interference contrast optics on a Zeiss Axioskop 2 plus at 400 × magnification.

### Data availability

All strains utilized are available from the *Caenorhabditis* Genetics Center (https://cbs.umn.edu/cgc/home) or from the authors by request.

## Results

We performed preliminary experiments with *C. briggsae him-8*; *myo*-2::*GFP X* hermaphrodites (strain PB192) that had been ostensibly purged of self-sperm by serial transfer over several days until embryo laying stopped, as done by Ragavapuram *et al.* (2016). The expectation was that roughly 15% of progeny from mating such purged hermaphrodites with *C. nigoni* males would be fertile males. Using the *C. nigoni* strain JU1422 in three different trials with 6–7 mothers each, 0/47, 3/50, and 0/116 progeny were male (1.4%).

Because Ragavapuram *et al.* (2016) used *C. nigoni* males of the African EG5268 strain, we next considered the possibility that the unexpectedly infrequent males obtained above were a strain effect. Using the purging approach, 7/66 progeny were male in the first experiment with EG5268 males (11%). Of these, two were extremely small and infertile, similar to those observed when F1 males have a *C. nigoni* X chromosome (Woodruff *et al.* 2010). Because *him-8* mothers produce nullo-X oocytes at an appreciable frequency, this is expected (Ragavapuram *et al.* 2016). Five others were GFP+, robust, and fertile, and thus candidates for rescued F1 hybrid males. However, plates bearing these putative hybrid males subsequently gave rise to vigorous populations of uniformly GFP+ animals. Individual virgin hermaphrodites isolated from these plates were invariably Him. This suggested that, despite purging, *C. briggsae* PB192 mothers could occasionally produce self-progeny after mating with *C. nigoni* males, perhaps via residual self-sperm that were resistant to purging. A second attempt to generate hybrid males with EG5268 sires produced three males, all GFP− and small.

The above preliminary crosses produced fertile males at a frequency lower than the expected 15%. They also indicated that complete purging in *C. briggsae* may be more difficult to achieve than had been previously appreciated. To ensure that all progeny being scored were interspecies hybrids, and to allow the use of younger, healthier mothers, self-sperm were ablated in PB192 by *Cbr-fog-3*(*RNAi*) via maternal injection (Chen *et al.* 2001). From over 2000 hybrid F1 embryos laid, 16 viable male adults were obtained (Table 1). These males had fully formed tails and exhibited mating behavior, but were GFP− and unusually small. Attempts to backcross them to their siblings failed to produce any embryos. Consistent with this apparent sterility, all of these males lacked a fully formed germline (often apparently completely absent).

**Table 1:** Phenotypes of progeny from *Cbr-him-8*; *myo-2*::*GFP*; *Cbr-fog-3(RNAi)* mothers mated to *C. nigoni* EG5268 wild-type males

Total Embryos	Total Viable Hybrid Progeny	Female Progeny	Male Progeny, GFP(−)	Male Progeny, GFP(+)
2045	1065 (52.1%)	1049 (51.3%)	16 (0.8%)	0 (0.0%)

GFP, green fluorescent protein.

The above results are consistent with all of the F1 hybrid males obtained in the crosses being derived from fertilization of a nullo-X *C. briggsae* oocyte by a *C. nigoni* male X-bearing sperm. This produces an X*_Cni_*O genotype known to produce sterile males (Woodruff *et al.* 2010). They also indicate a lack of any viability or fertility of F1 X*_Cbr_*O males, contrary to the interpretation of Ragavapuram *et al.* (2016). To be sure that the germline feminization of P0 *C. briggsae* hermaphrodites by RNAi did not suppress normal fertility in their sons, we verified that male offspring of *Cbr-fog-3*(*RNAi*) Fog mothers have normal fertility with conspecific matings (data not shown).

## Discussion

In both *C. elegans* and *C. briggsae*, loss of *him-8* function specifically impairs X chromosome pairing (Phillips *et al.* 2005; Wei *et al.* 2014), and unpaired *C. elegans* chromosomes are subject to meiotic silencing (Bean *et al.* 2004). In addition, hemizygosity of the X chromosome is thought to underlie Haldane’s rule in male-heterogametic systems. These observations led Ragavapuram *et al.* (2016) to hypothesize that *Cbr-him-8* mutant hermaphrodites produce X-bearing oocytes with altered X-linked gene expression that rescues X*_Cbr_*O F1 viability. As plausible as this mechanism is, we were unable to replicate the rescue of these males in our own hybrid crosses when all possibility of selfing was eliminated via *Cbr-fog-3*(*RNAi*). The rare sterile males we did observe resulted from a *C. nigoni* X-bearing sperm fertilizing a nullo-X oocyte of the *C. briggsae* hermaphrodite, which often occurs in the *Cbr-him-8* PB192 strain.

It is conceivable that the use of *Cbr-fog-3*(*RNAi*) somehow blocks suppression of X*_Cbr_*O lethality that would otherwise be provided by the *Cbr-him-8* mutations. However, we also saw few or no fertile males in crosses with purged hermaphrodites lacking this treatment, and those that were produced appeared not to be interspecies hybrids. Therefore, we conclude that the previous report of X*_Cbr_*O hybrid male rescue was premature, and may be the result of incomplete purging of *C. briggsae* mothers. Perhaps importantly, the PB192 strain produces male self-progeny at a rate similar to that reported by Ragavapuram *et al.* (2016) as F1 hybrids.

We see some evidence for cryptic retention of a small number of self-sperm in putatively purged *C. briggsae* hermaphrodites. This may occur if sperm-derived major sperm protein-mediated signaling is insufficient to stimulate ovulation (Miller *et al.* 2001). Subsequent mating with *C. nigoni* males could stimulate resumption of ovulation, allowing the remaining conspecific sperm to be used. In addition, *C. briggsae* X-bearing sperm contributed by males are preferentially used by hermaphrodites over those lacking an X (LaMunyon and Ward 1997), such that the last sperm used are highly enriched for nullo-X gametes. Because *Cbr-him-8* hermaphrodites produce an unusual number of nullo-X self-sperm (Wei *et al.* 2014), a similar mechanism could produce *C. briggsae* PB192 XO males that are both fertile and GFP+ from only a handful of residual self-sperm. However, this preferential fertilization by X-bearing sperm is not seen with another *C. briggsae* mutation that induces nullo-X self-sperm at rates comparable to the *him-8* mutations employed here (LaMunyon and Ward 1997). Whatever the mechanism involved, the production of rare selfed progeny from nominally purged animals underscores the need for caution.

It remains possible that, under some conditions, fertile *C. briggsae* × *C. nigoni* hybrid males may yet be produced. If so, it will be crucial to demonstrate their hybrid nature by genotyping assays. Sufficient genome data now exist for *C. briggsae* (Ross *et al.* 2011) and *C. nigoni* (Li *et al.* 2016) to make such assays, such as PCR amplification of indel polymorphisms (Koboldt *et al.* 2010), rapid and inexpensive.
